# Finite-Time Attitude Stabilization Adaptive Control for Spacecraft with Actuator Dynamics

**DOI:** 10.3390/s19245568

**Published:** 2019-12-16

**Authors:** Chunbao Wang, Dong Ye, Zhongcheng Mu, Zhaowei Sun, Shufan Wu

**Affiliations:** 1 Research Center of Satellite Technology, Harbin Institute of Technology, Harbin 150080, China; wangchunbao@hit.edu.cn (C.W.); yed@hit.edu.cn (D.Y.); sunzhaowei@hit.edu.cn (Z.S.); 2 School of Aeronautics and Astronautics, Shanghai Jiao Tong University, Shanghai 200240, China; shufan.wu@sjtu.edu.cn

**Keywords:** attitude stabilization, actuator dynamics, double fast terminal sliding mode, adaptive law

## Abstract

For the attitude stabilization of spacecraft with actuator dynamics, this paper proposed a finite-time control law. Firstly, the dynamic property of the actuator is analyzed by an example. Then, a basic control law is derived to achieve the finite-time stability using the double fast terminal sliding mode manifold. When there is no prior knowledge of time matrix of the actuator, an adaptive law is proposed to estimate the unknown information. An adaptive control law is derived to guarantee the finite-time convergence of the attitude, and a Lyapunov-based analysis is provided. Finally, simulations are carried out to demonstrate the effectiveness of the proposed control law to the attitude stabilization with the actuator dynamics. The results show that the high-precision attitude control performance can be achieved by the proposed scheme.

## 1. Introduction

Spacecraft mission success often relies on performance of the attitude control system, which consists of different types of actuators, such as thrusters and the reaction wheel, etc. In the attitude control problem, the actuator is often assumed to be the ideal dynamic [1,2,3,4] and must be able to deliver the exact torque desired by the controller immediately, which does not always conform to the actual situation [5]. Nevertheless, if the actuator dynamics are neglected for high-precision attitude control, it may have an impact on the control performance. Therefore, it makes sense to study the attitude stabilization control while taking into account the actuator dynamics. 

There are many studies on spacecraft attitude control, and several control methods have been developed, such as backstepping [6,7] and sliding mode control [1,2,3,4], etc. Hu [1] proposed a novel time-varying fast terminal sliding mode manifold to derive a finite-time controller in the presence of external disturbance, input saturation, and inertia uncertainty. A modification matrix is introduced to maximize the torque envelopes with all functional actuators. Song [2] proposed a fast terminal sliding mode control scheme with double closed loops. The inner-loop controller guaranteed the tracking of the angular velocity, and the outer-loop controller guaranteed the tracking of the attitude angle. Simulations showed the robustness for the external disturbance and finite-time stability performance. Zou [4] designed a finite-time attitude-tracking controller combing terminal sliding mode and Chebyshev neural network. The nonlinear part in controller would be estimated through the network, and the stability was proven using Lyapunov stability. Xiao [7] addressed the fault-tolerant attitude tracking problem without angular velocity measurements. By the terminal sliding mode observer, the state could be reconstructed and a backstepping-like controller was derived without the requirement of fault detection and diagnosis mechanism. Han [8] considered two types of actuator failures, including a gain fault and a deviation fault, and developed an adaptive fault-tolerant controller. The time-dependent parameters were introduced to weight the components of the feedback control, and to improve the responsiveness of attitude error. Ruiter [9] proposed an adaptive controller with input saturation for spacecraft attitude tracking. An adaptive law was designed to estimate unknown parameterizable disturbance. In addition, a control law, consisting of a feedforward and a feedback component, was given to compensate the disturbance. In [10], a finite-time controller was proposed using the integral second order sliding mode manifold. An adaptive law was proposed to eliminate the need of prior knowledge of uncertainty and controller gains, which improved the control robustness. The stability proof was completed by the Lyapunov approach and the negative homogeneity approach. Thakur [11] developed the attitude tracking problem with uncertain time-varying inertia parameters, which was divided into unknown rigid components and the nonrigid time-varying components. The adaptive controller was proposed for the time-dependent and state-dependent inertia, input-dependent inertia, respectively. In addition, a smooth projection scheme was proposed to prevent drifting of the parameter estimates. In [12], the finite-time attitude control for flexible spacecraft was addressed. A controller was proposed using the fast nonsingular terminal sliding mode manifold with a weighted homogeneous extended state observer estimating unknown disturbance. A new path planning with the input technique was developed to reduce vibrations. Simulation results showed the well attitude convergence performance and the well vibration attenuation performance. 

For the attitude control considering actuator dynamics, Hu [6,13] considered the attitude control problem with flexible appendages and flywheel dynamics. The controller, in the form of the input voltage of the reaction wheel, was derived using the sliding mode technique. An adaptive version of the control law was designed to adapt the unknown disturbance. In addition, strain rate feedback method was used to actively suppress the induced vibration. Through simulations, the requirement of high-precision control could be met using the proposed scheme. In [5], the dynamic behavior of different actuators was investigated using functional description and theoretical frameworks. A generalized actuator dynamics was given, including a first-order and second-order linear approximation. When deriving a controller, the proposed models were considered to enable increased control precision.

Although there are massive studies on attitude stabilization, most neglect the actuator dynamics [1,2,3,4] or only take into account a specified actuator [6,13]. Nevertheless, due to its dynamics in practice, the actuator cannot deliver the desired torque provided by the controller instantaneously. This will degrade the control performance for the high-precision attitude stabilization. In addition, with most actuators performing similar dynamical behavior, it is useful to establish a general dynamic model of actuator. For this problem, this paper studies the attitude stabilization control with a general dynamics of the actuator. A basic control law and an adaptive control law are derived using the double fast terminal sliding mode (FTSM) manifold. The finite-time stabilization proof is completed by the Lyapunov-based theory.

The paper is organized as follows: In Section 2, the attitude dynamics and kinematics with the actuator dynamics are introduced. Then, a basic finite-time control law and a finite-time adaptive control law are provided using the double fast terminal sliding mode in Section 3. Finally, several simulations are performed and we conclude the paper.

## 2. Mathematical Model with Actuator Dynamics

### 2.1. Notation

For a diagonal matrix $A=\left\{a_{ii}\right\}\in R^{n\times n}$, let V(A) denote the vectorization of the matrix, namely, $V(A)={\left[\begin{array}{cccc} a_{11} & a_{22} & \ldots & a_{nn} \end{array}\right]}^{T}$.

Let $\lambda_{\max}\left(\cdot \right)$, $\lambda_{\min}\left(\cdot \right)$ denote the maximum and the minimum eigenvalue of a matrix, respectively.

### 2.2. Attitude Dynamics and Kinematics

To describe the spacecraft attitude dynamics, we establish coordinate frames as follows: the inertial-reference frame I and the body-fixed frame B. The inertial-reference frame is chosen such that its origin is the center of the earth, the x-axis points toward Vernal Equinox, the z axis is aligned with the rotation axis of the Earth, and the y-axis competes the right-hand rule. The body frame B is chosen such that its origin is the center of mass of the spacecraft and the three axes coincide with the principal axes of the spacecraft. 

In this paper, the quaternion, due to its nonsingularity, is used to represent the spacecraft attitude mapping from the inertial-reference frame to the body-fixed frame. The attitude kinematics and dynamics can be expressed as [14]:

(1) $$\begin{array}{l} {\dot{q}}_{0}=-\frac{1}{2}q^{T}w\\ \dot{q}=\frac{1}{2}A(Q)w\\ J\dot{w}+w^{\times }Jw=u+d \end{array}$$

where $A(Q)=q_{0}I_{3}+q^{\times }$ with $q_{0}$ and q denote the scalar and vector components of the unit quaternion $Q={\left[\begin{array}{cc} q_{0} & q \end{array}\right]}^{T}\in R\times R^{3}$, and satisfy the constrain $q_{0}^{2}+q^{T}q=1$. J is the inertia matrix of the spacecraft. $w\in R^{3}$ is the angular velocity of the spacecraft with respect to the inertial-reference frame and expressed in the body-fixed frame. $u\in R^{3}$ is the actual control torque of the actuator, and $d\in R^{3}$ is the total external disturbance. For a vector $a={\left[\begin{array}{ccc} a_{1} & a_{2} & a_{3} \end{array}\right]}^{T}\in R^{3}$, $a^{\times }\in R^{3\times 3}$ denotes the vector cross-product operator defined by

$$a^{\times }=\left[\begin{array}{ccc} 0 & -a_{3} & a_{2}\\ a_{3} & 0 & -a_{1}\\ -a_{2} & a_{1} & 0 \end{array}\right]$$

For the attitude stabilization control, defining the desired unit quaternion as $Q_{d}={\left[\begin{array}{cc} q_{d0} & q_{d} \end{array}\right]}^{T}$, and the desired angular velocity as $w_{d}=0$, the errors of the quaternion and the angular velocity are given as follows:

(2) $$\begin{array}{l} Q_{e}=Q_{d}^{-1}⊗Q=\left[\begin{array}{c} q_{d}^{T}q+q_{d0}q_{0}\\ q_{d0}q-q_{0}q_{d}-q_{d}^{\times }q \end{array}\right]\\ w_{e}=w-w_{d}=w \end{array}$$

where ⊗ represent the quaternion multiplication.

Differentiating Equation (2), the attitude error dynamics and kinematics can be written as [12]:

(3) $$\begin{array}{l} {\dot{q}}_{0e}=-\frac{1}{2}q_{e}^{T}w\\ {\dot{q}}_{e}=\frac{1}{2}A(Q_{e})w\\ J\dot{w}+w^{\times }Jw=u+d \end{array}$$

### 2.3. Actuator Dynamics

For the attitude error dynamics (3), the actuator is considered to be ideal, which indicates that the actuator can deliver the exact torque desired by the attitude controller at a specified time [5]. However, the actuator has its own dynamics in practice, which may impact the controller performance. From [5], we know that there exist similar dynamical behaviors in different actuators despite of different dynamic models. Thus, a linear general actuator dynamic model is presented as:

(4) $$T\dot{u}+u=v$$

where T is a diagonal matrix with positive time constant, namely, $T=T^{T}>0$. v is the desired control torque of the actuator.

**Figure 1:**
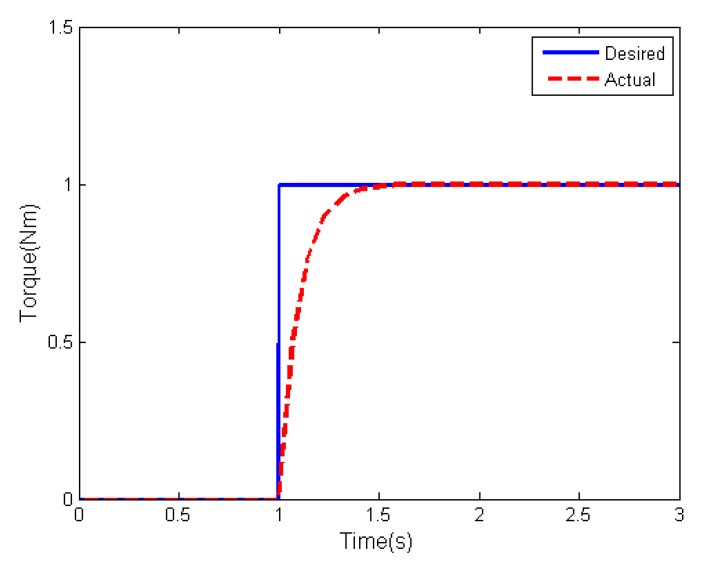
Actuator response to desired control torque.

To show the dynamic performance of the actuator, we consider the one-axis dynamics with the time constant being $T_{i}=0.1s$. At t=1s, a desired step torque is delivered to the actuator, then the response of the actuator is depicted in Figure 1. It is seen that the actuator with dynamics cannot respond the desired torque instantaneously, which may impact the high-precision attitude stabilization performance. Thus, it makes sense to study the attitude control with taking into account the dynamic property of actuator.

Through the above analysis, the complete attitude error dynamics with actuator dynamics can be expressed as:

(5) $$\begin{array}{l} {\dot{q}}_{e0}=-\frac{1}{2}q_{e}^{T}w\\ {\dot{q}}_{e}=\frac{1}{2}A(Q_{e})w\\ J\dot{w}+w^{\times }Jw=u+d\\ T\dot{u}+u=v \end{array}$$

### 2.4. Assumptions and Lemmas

For the development of the controller and the proof later, some assumptions and lemmas are given as follows.

**Assumption** **1.** *The scalar component of the error quaternion is always more than zero, namely,* $q_{e0}>0$ *.*

**Assumption** **2.** *The external disturbance and its rate are bounded and their bounds are known, namely,* $\left\|d\right\|\leq d_{\max}$ *,* $\left\|\dot{d}\right\|\leq {\dot{d}}_{\max}$ *.*

**Assumption** **3.** *The inertia matrix* J *and the time matrix* T *are bounded symmetric positive definite matrices.*

**Lemma** **1** [15,16]**.** *For the continuous system* $\dot{x}=f(x)$*,* f(0)=0*,* $x\in R^{n}$*. Suppose there is a positive function* $V:R^{n}\to R^{n}$*, positive numbers* a>0*,* b>0*, and* m∈(0,1) *defined on* $U_{0}\subseteq R^{n}$ *in the open neighborhood of the origin such that* $\begin{array}{cc} \dot{V}(x)+aV(x)+bV^{m}(x)\leq 0 & x\in U_{0}\backslash \{0\} \end{array}$*. Then the origin is a finite-time stable equilibrium. If* $U_{0}=R^{n}$*, the origin is a globally finite-time stable equilibrium. And the finite time to reaching the origin t depending on the initial condition* $x(0)=x_{0}$ *is*

$$t\leq \frac{1}{a(1-m)}\ln\frac{aV^{1-m}(x_{0})+b}{b}$$

**Lemma** **2** [2]**.** *Assume* $a_{1}$*,* $a_{2}$*, …,* $a_{n}$ *are all real numbers, then the following inequality holds*

$$\left|a_{1}\right|+\left|a_{2}\right|+\cdots +\left|a_{n}\right|\geq {\left(a_{1}^{2}+a_{2}^{2}+\cdots a_{n}^{2}\right)}^{\frac{1}{2}}$$

## 3. Attitude Control Law with Actuator Dynamics Design

In this section, we show the development of a control law to solve the attitude stabilization with the actuator dynamics using the sliding mode manifold. Nevertheless, a sliding mode manifold needs to be differentiated secondly until the desired torque appears as the control term, then the conventional sliding mode control is not applicable. Thus, a second sliding manifold is introduced to asymptotically stabilize the first sliding manifold with a guaranteed quality [17]. First, a basic double FTSM control law is designed, assuming a prior knowledge of the time matrix of the actuator and the angular acceleration. Then, an adaptive double FTSM control law is designed without relying on prior knowledge.

### 3.1. Basic Double FTSM Control Law Design

**Figure 2:**
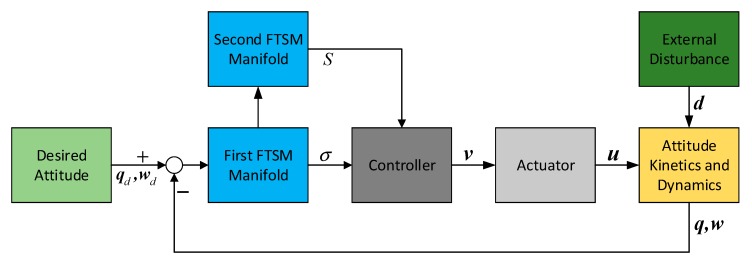
Structure of basic double fast terminal sliding mode (FTSM) control system.

For the attitude stabilization with actuator dynamics, the basic double FTSM manifold is introduced to design the control law [17,18]. The structure of the attitude stabilization system with basic double FTSM can be illustrated in Figure 2.

The first FTSM manifold is defined as:

(6) $$\sigma =w+\alpha_{1}q_{e}+\alpha_{2}q_{e}^{\frac{a_{1}}{a_{2}}}$$

where $\alpha_{1}>0$, $\alpha_{2}>0$, and $a_{1}$, $a_{2}$ are positive odd integers satisfying $0<\frac{a_{1}}{a_{2}}<1$. 

Taking the first-order and second-order derivatives of Equation (6), we have:

(7) $$\begin{array}{l} \dot{\sigma }=\dot{w}+\alpha_{1}{\dot{q}}_{e}+\alpha_{2}\frac{a_{1}}{a_{2}}diag\left\{q_{e}^{\frac{a_{1}}{a_{2}}-1}\right\}{\dot{q}}_{e}\\ \ddot{\sigma }=\ddot{w}+\alpha_{1}{\ddot{q}}_{e}+\alpha_{2}\left[\frac{a_{1}}{a_{2}}diag\left\{q_{e}^{\frac{a_{1}}{a_{2}}-1}\right\}{\ddot{q}}_{e}+\frac{a_{1}}{a_{2}}\left(\frac{a_{1}}{a_{2}}-1\right)diag\left\{q_{e}^{\frac{a_{1}}{a_{2}}-2}\right\}{\dot{q}}_{e}^{2}\right] \end{array}$$

It implies that, if $q_{e}=0$ and ${\dot{q}}_{e}\neq 0$, then the singularity of the sliding manifold will occur due to $\frac{a_{1}}{a_{2}}-1<0$. To avoid this problem, the first-order derivative of FTSM is modified as:

(8) $$\dot{\sigma }=\dot{w}+\alpha_{1}{\dot{q}}_{e}+\alpha_{2}a^{\prime }(q_{e})$$

with $a^{\prime }(q_{e})\in R^{3}$ being:

$$a_{j}^{\prime }(q_{ej})=\{\begin{array}{cc} \frac{a_{1}}{a_{2}}q_{ej}^{\frac{a_{1}}{a_{2}}-1}{\dot{q}}_{ej} & \mathrm{if}\;{\dot{q}}_{ej}\neq 0,\left|q_{ej}\right|\geq \mu_{1}\\ \frac{a_{1}}{a_{2}}\mu_{1}^{\frac{a_{1}}{a_{2}}-1}{\dot{q}}_{e} & \mathrm{if}\;{\dot{q}}_{ej}\neq 0,\left|q_{ej}\right|<\mu_{1}\\ 0 & {\dot{q}}_{ej}=0 \end{array}$$

where $a_{j}^{\prime }(q_{ej})$ is the jth component of $a^{\prime }(q_{e})$ and $\mu_{1}$ is a small positive constant.

The second time derivate is obtained:

(9) $$\ddot{\sigma }=\ddot{w}+\alpha_{1}{\ddot{q}}_{e}+\alpha_{2}a^{\prime \prime }(q_{e})$$

with

$$a_{j}^{\prime \prime }(q_{ej})=\{\begin{array}{cc} \frac{a_{1}}{a_{2}}q_{ej}^{\frac{a_{1}}{a_{2}}-1}{\ddot{q}}_{ej}+\frac{a_{1}}{a_{2}}\left(\frac{a_{1}}{a_{2}}-1\right)q_{ej}^{\frac{a_{1}}{a_{2}}-2}{\dot{q}}_{ej}^{2} & \mathrm{if}\;{\dot{q}}_{ej}\neq 0,\left|q_{ej}\right|\geq \mu_{1}\\ \frac{a_{1}}{a_{2}}\mu^{\frac{a_{1}}{a_{2}}-1}{\ddot{q}}_{ej}+\frac{a_{1}}{a_{2}}\left(\frac{a_{1}}{a_{2}}-1\right)\mu^{\frac{a_{1}}{a_{2}}-2}{\dot{q}}_{ej}^{2} & \mathrm{if}\;{\dot{q}}_{ej}\neq 0,\left|q_{ej}\right|<\mu_{1}\\ 0 & {\dot{q}}_{ej}=0 \end{array}$$

Furthermore, we define the second modified FTSM manifold as:

(10) $$S=\dot{\sigma }+\beta_{1}\sigma +\beta_{2}\sigma^{\frac{b_{1}}{b_{2}}}$$

with $\beta_{1}>0$, $\beta_{2}>0$, and $b_{1}$, $b_{2}$ are positive odd integers satisfying $0<\frac{b_{1}}{b_{2}}<1$. Differentiating Equation (10) and modifying the derivative, we have:

(11) $$\dot{S}=\ddot{\sigma }+\beta_{1}\dot{\sigma }+\beta_{2}b^{\prime }(\sigma )$$

with $b^{\prime }(\sigma )\in R^{3}$ being:

(12) $$b_{j}^{\prime }(\sigma_{j})=\{\begin{array}{cc} \frac{b_{1}}{b_{2}}\sigma_{j}^{\frac{b_{1}}{b_{2}}-1}{\dot{\sigma }}_{j} & \mathrm{if}\;{\dot{\sigma }}_{j}\neq 0,\left|\sigma_{j}\right|\geq \mu_{2}\\ \frac{b_{1}}{b_{2}}\mu_{2}^{\frac{b_{1}}{b_{2}}-1}{\dot{\sigma }}_{j} & \mathrm{if}\;{\dot{\sigma }}_{j}\neq 0,\left|\sigma_{j}\right|<\mu_{2}\\ 0 & {\dot{\sigma }}_{j}=0 \end{array}$$

where $b_{j}^{\prime }(\sigma_{j})$ is the jth component of $b^{\prime }(\sigma )$ and $\mu_{2}$ is a small positive constant.

By multiplying a matrix, defined as P≜TJ, on both sides of (11) and then combining with Equations (5) and (9), we have:

(13) $$\begin{array}{ll} P\dot{S} & =P\left[J^{-1}T^{-1}v+J^{-1}\left(-{\dot{w}}^{\times }Jw-w^{\times }J\dot{w}-T^{-1}u+\dot{d}\right)+\alpha_{1}{\ddot{q}}_{e}+\alpha_{2}a^{\prime \prime }(q_{e})+\beta_{1}\dot{\sigma }+\beta_{2}b^{\prime }(\sigma )\right]\\ & =v+TJ\left[J^{-1}\left(-{\dot{w}}^{\times }Jw-w^{\times }J\dot{w}\right)+\alpha_{1}{\ddot{q}}_{e}+\alpha_{2}a^{\prime \prime }(q_{e})+\beta_{1}\dot{\sigma }+\beta_{2}b^{\prime }(\sigma )+J^{-1}\dot{d}\right]-u \end{array}$$

Therefore, the attitude stabilization control law with the actuator dynamics can be derived as

(14) $$v=-T\left[\begin{array}{l} \left(-{\dot{w}}^{\times }Jw-w^{\times }J\dot{w}\right)+\\ J\left(\alpha_{1}{\ddot{q}}_{e}+\alpha_{2}a^{\prime \prime }(q_{e})+\beta_{1}\dot{\sigma }+\beta_{2}b^{\prime }(\sigma )\right) \end{array}\right]+u-K_{1}S-K_{2}\frac{S}{\left\|S\right\|}$$

where $K_{1}$, $K_{2}$ are positive scalers satisfying

(15) $$K_{1}>0,K_{2}=\max\left\{\lambda_{\max}\left(T\right){\dot{d}}_{\max}\right\}+\zeta$$

with ζ being a positive scalar.

**Theorem** **1.** *Consider the attitude error dynamics (5) and the control law (14). If the assumptions 1–2 are satisfied, then the closed-loop system is stabilized in finite time, i.e.,* $q_{e}\to 0$ *,* w→0 *in finite time.*

**Proof.**  Please see Appendix A. □

### 3.2. Adaptive Double FTSM Control Law Design

**Figure 3:**
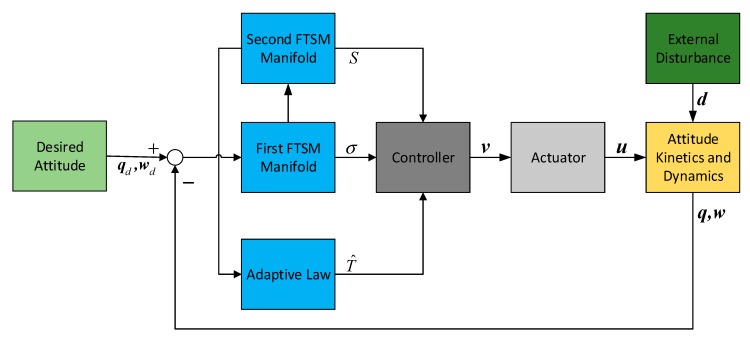
Structure of adaptive double FTSM control system.

For the proposed control law (14), the time matrix T is assumed to be known in advance. In practice, such information is not always available due to the nonmeasurability of the actuator dynamics. Thus, to avoid the requirements of a prior knowledge, an adaptive law is introduced to estimate the time matrix T. The structure of the attitude stabilization system with adaptive double FTSM can be illustrated in Figure 3.

Then, define the actual value of the time matrix as T, and the estimation as $\hat{T}$, then the estimation error can be given as $\tilde{T}=T-\hat{T}$, and satisfies $\dot{\tilde{T}}=-\dot{\hat{T}}$ with fixed T. Thus, the adaptive control law can be given as:

(16) $$v=-\hat{T}\left[\begin{array}{l} (-{\dot{\hat{w}}}^{\times }Jw-w^{\times }J\dot{\hat{w}})+\\ J(\alpha_{1}{\ddot{q}}_{e}+\alpha_{2}a^{\prime \prime }(q_{e})+\beta_{1}\dot{\sigma }+\beta_{2}b^{\prime }(\sigma )) \end{array}\right]+u-K_{1}S-K_{2}\frac{S}{\left\|S\right\|}$$

where $K_{1}$, $K_{2}$ are positive scalers satisfying:

(17) $$K_{1}>0,K_{2}=\max\left\{\lambda_{\max}\left(T\right){\dot{d}}_{\max}\right\}$$

The adaptive law of the time matrix is chosen as:

(18) $$V(\dot{\hat{T}})=M^{-1}diag\left\{\begin{array}{l} \left(-{\dot{w}}^{\times }Jw-w^{\times }J\dot{w}\right)+\\ J\left(\alpha_{1}{\ddot{q}}_{e}+\alpha_{2}a^{\prime \prime }(q_{e})+\beta_{1}\dot{\sigma }+\beta_{2}b^{\prime }(\sigma )\right) \end{array}\right\}S-M^{-1}V(\hat{T})$$

where M is a positive definite diagonal matrix.

**Theorem** **2.** *Consider the error attitude dynamics (5), the adaptive control law (16) and the adaptive law (18). If the assumptions 1–2 are satisfied, the sliding mode* S *,* σ *converge to the neighborhood of* S=0 *and* σ=0 *in finite time. Furthermore, the attitude and the angular velocity converges to the regions*

$$\left|q_{ej}\right|\leq \Delta {,}^{}\left|w_{j}\right|\leq 3\delta$$

*in finite time, respectively, where* Δ *and* δ *will be given later.*

**Proof.**  Please see Appendix B. □

**Remark** **1.** *The proposed control law is discontinuous, because of the function* $\frac{S}{\left\|S\right\|}$ *, which may cause the undesirable chattering phenomenon in sliding mode. To alleviate this problem, the discontinuous function is replaced with the continuous function* $\frac{S}{\left\|S\right\|+\varepsilon }$ *, where* ε *is a small positive scalar [19].*

**Remark** **2.** *As seen in Equation (18), the adaptive law proposed is in a feedback form. When the attitude quaternion and the angular velocity converge to origin, the first term of the right side will be zero and then the adaptive law (18) can be rewritten as:*

(19) $$V(\dot{\hat{T}})=-M^{-1}V(\hat{T})$$

Because ***M*** is a positive definite matrix, the time matrix has a feedback form with negative eigenvalues. Thus, the time estimation will converge to zero ultimately.

**Remark** **3.** *Although the control law is derived with the first-order dynamics of the actuator, the proposed control law can be extended to the second-order dynamics scheme. This is because the second-order dynamic model can be rewritten as two first-order dynamic models, as expressed in [5], and then the proposed FTSM approach is also applicable.*

## 4. Numerical Simulations

To demonstrate the effectiveness of the proposed control law to the attitude stabilization with the actuator dynamics, several simulations are carried out. Initial conditions of spacecraft are listed as follows.

The mass moment of inertia matrix of the spacecraft is

$$J=\left[\begin{array}{ccc} 15 & 1 & 0.5\\ 1 & 15 & 0.8\\ 0.5 & 0.8 & 15 \end{array}\right]\mathrm{kg}\cdot m^{2}$$

The nominal time matrix of the actuator is assumed as $T=diag\left\{\left[\begin{array}{ccc} 0.1 & 0.1 & 0.1 \end{array}\right]\right\}$. The initial attitude quaternion is given as $Q={\left[\begin{array}{cccc} 0.911 & 0.2 & -0.2 & 0.3 \end{array}\right]}^{T}$, and the angular velocity is $w=\left[\begin{array}{ccc} -0.034 & 0.037 & -0.047 \end{array}\right]\mathrm{rad}/s$. The desired quaternion is chosen as $Q_{d}={\left[\begin{array}{cccc} 1 & 0 & 0 & 0 \end{array}\right]}^{T}$. The external disturbance is assumed to be

$$d=\left[\begin{array}{c} 5+\sin\left(0.5t\right)+\mathrm{randn}\\ 1-10\cos\left(0.5t\right)+\mathrm{randn}\\ -3+2\sin\left(0.2t+\pi /3\right)+\mathrm{randn} \end{array}\right]\times 10^{-4}\mathrm{Nm}$$

The maximum torque of the actuator is assumed as 0.2 nm.

The parameters in the control law are chosen as follows: $a_{1}=b_{1}=5$, $a_{2}=b_{2}=7$, $\alpha_{1}=\beta_{1}=0.8$, $\alpha_{2}=\beta_{2}=0.01$, $\mu_{1}=\mu_{2}=0.01$, $K_{1}=K_{2}=0.05$; and the parameter in adaptive law is chosen as follows: $M=diag\left\{\left[\begin{array}{ccc} 7 & 7 & 7 \end{array}\right]\right\}$.

(1) Basic Double FTSM Control Law 

**Figure 4:**
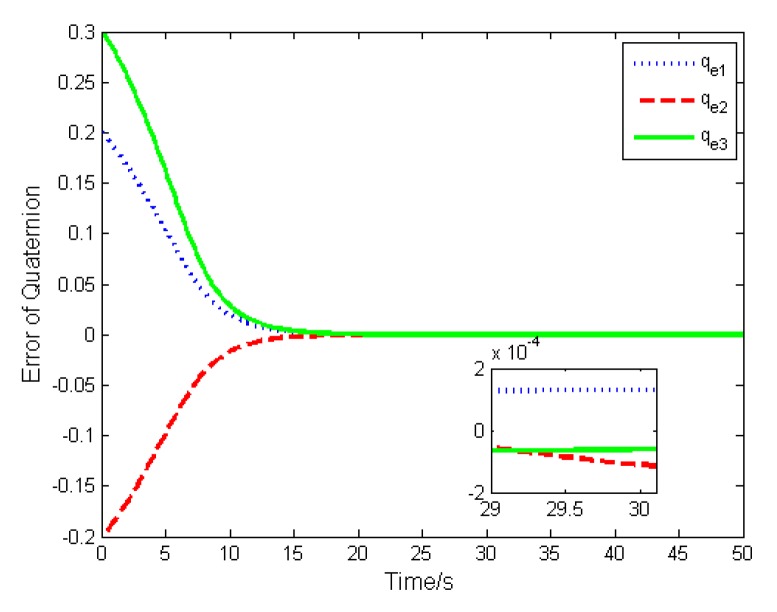
Error of attitude quaternion.

**Figure 5:**
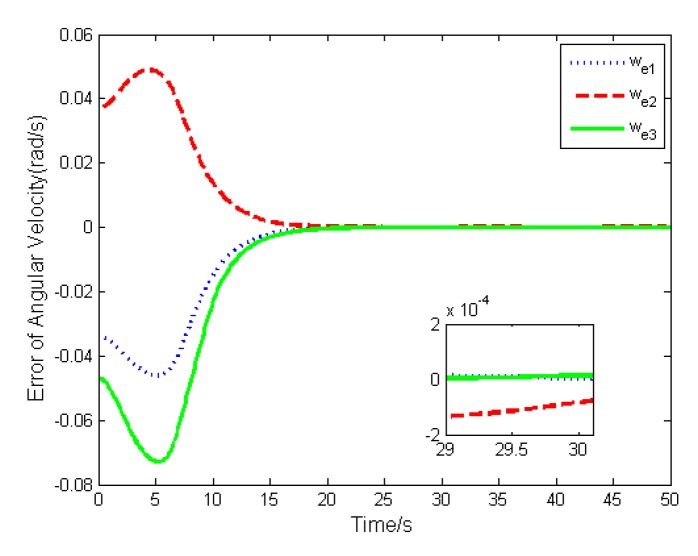
Error of angular velocity.

In this case, the basic double FTSM control law (14) is used to stabilize the attitude. The errors of the attitude quaternion and the angular velocity are given in Figure 4 and Figure 5. It is seen that the quaternion and the angular velocity achieve stabilization within 20 s, and the steady-state errors achieve $10^{-4}$ orders of magnitude, which meets the requirement of the highly accurate control. 

**Figure 6:**
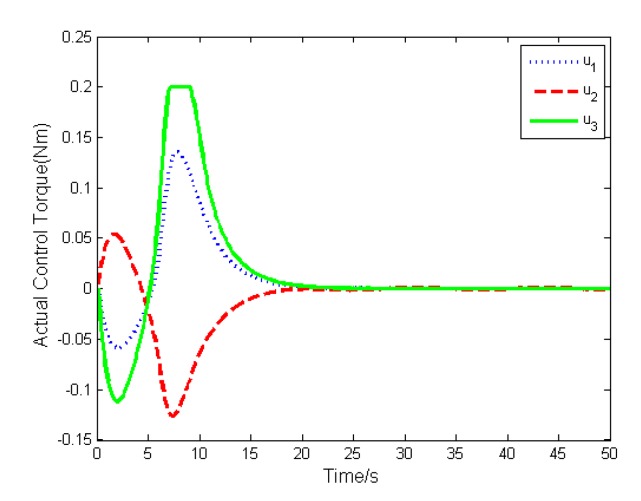
Actual torque of actuator.

**Figure 7:**
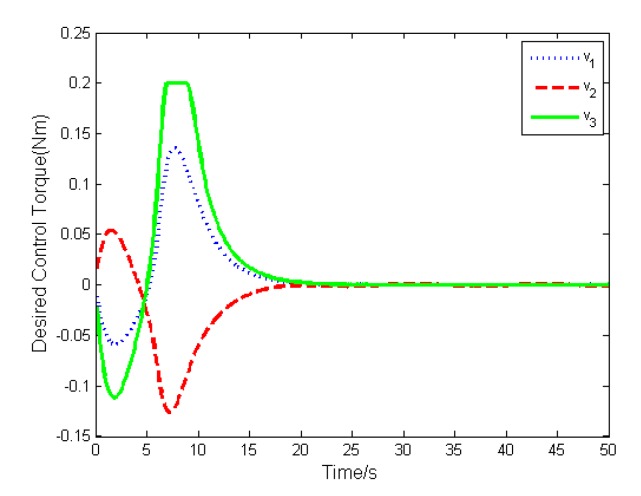
Desired torque of actuator.

**Figure 8:**
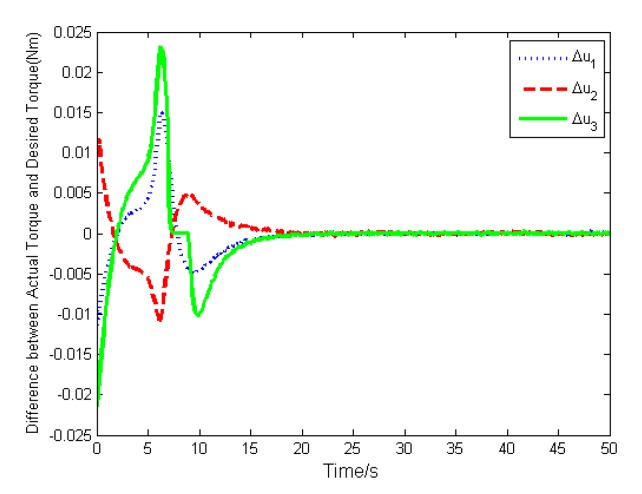
Torque difference.

In addition, the actual torque and the desired torque of the actuator are shown in Figure 6 and Figure 7. The results show that both torque will converge to zero with the attitude stabilization. To illustrate the difference between the actual torque and the desired torque, we define their difference as Δu=v−u, and depict it in Figure 8. Because the actuator cannot deliver the exact torque instantaneously, there exists difference between the actual torque and the desired torque, which will impact the high-precision attitude stabilization. 

(2) Adaptive Double FTSM Control Law

To illustrate the effectiveness of the adaptive double FTSM scheme defined in (16), the attitude stabilization problem without a prior knowledge is simulated. We assume that the initial estimation of the time matrix is $diag\left\{\left[\begin{array}{ccc} 0.09 & 0.09 & 0.09 \end{array}\right]\right\}$.

**Figure 9:**
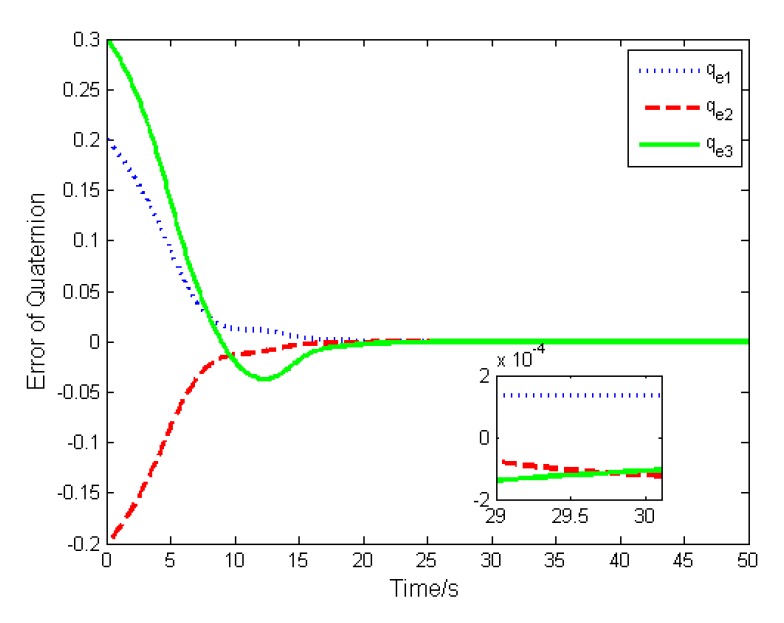
Error of attitude quaternion.

**Figure 10:**
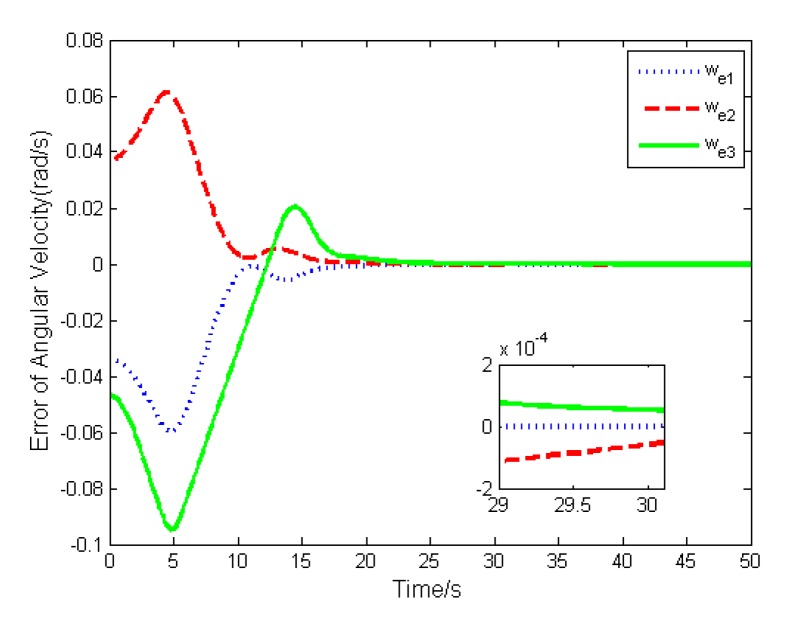
Error of angular velocity.

**Figure 11:**
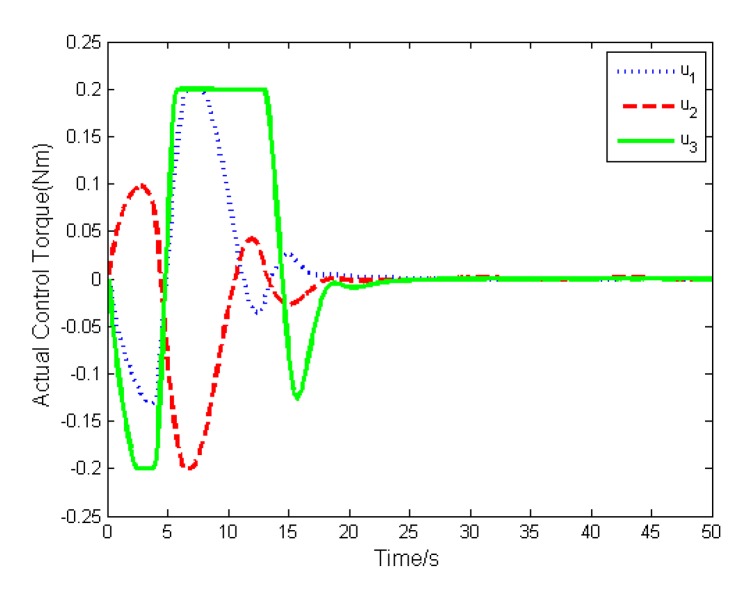
Actual torque of actuator.

**Figure 12:**
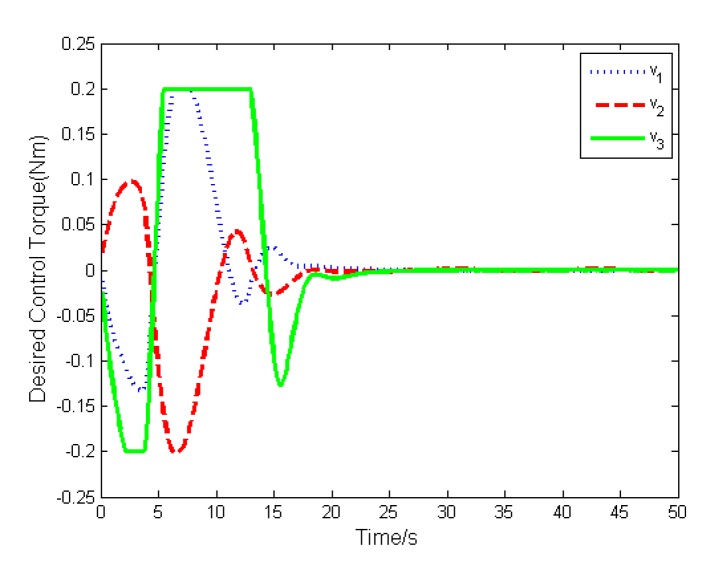
Desired torque of actuator.

**Figure 13:**
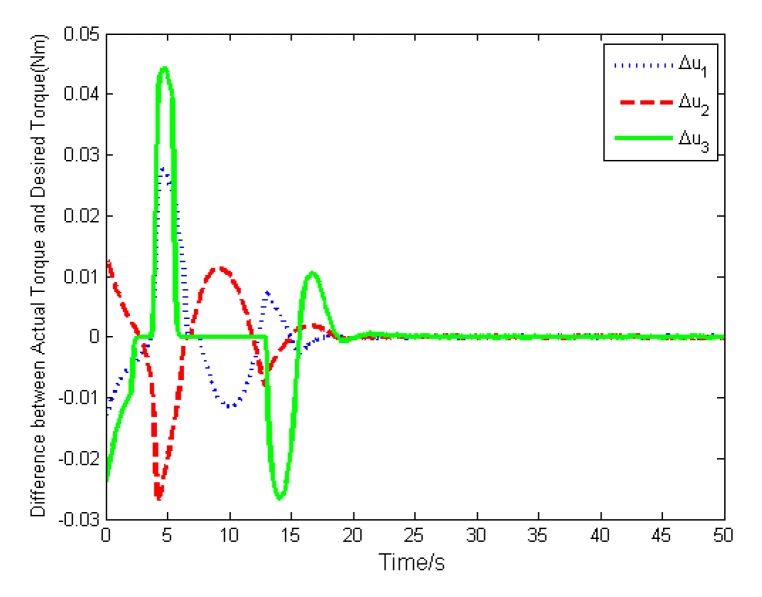
Torque difference.

**Figure 14:**
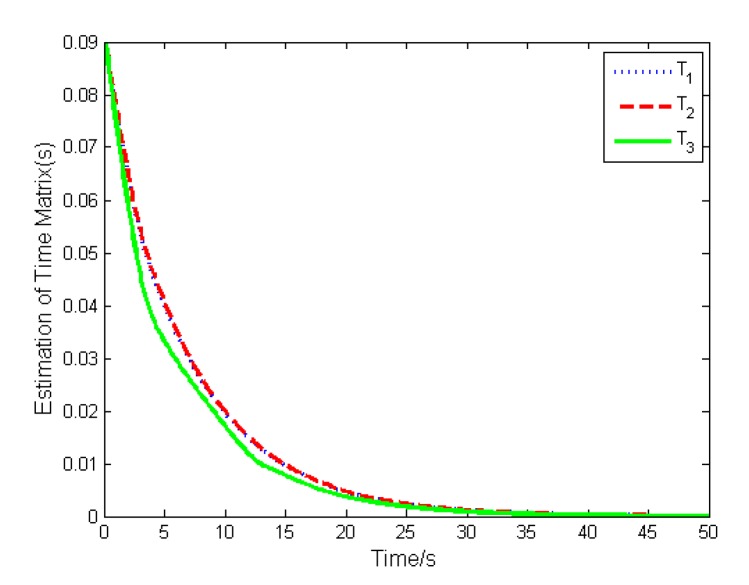
Estimation of time matrix.

The simulation results are depicted in Figure 9, Figure 10, Figure 11, Figure 12, Figure 13 and Figure 14, from which we can find that the stabilization time is longer than that using basic double FTSM. This is because the prior knowledge of the time matrix of the actuator is absent. Nevertheless, the steady-state errors of the quaternion and the angular velocity also achieve high precision with 10–6 orders of magnitude, which verifies the efficacy of the proposed control law. 

The torque difference is shown in Figure 13, from which we can find that the maximal torque difference is larger than that of case (1) due to no prior knowledge of time matrix. The estimation of the time matrix is depicted in Figure 14. As expressed in Remark 2, when the attitude quaternion and the angular velocity achieve stabilization, the estimation of the time matrix will converge to zero. The result shows that the estimation is bounded, which is the estimation performance.

## 5. Conclusions

This paper investigates the spacecraft attitude stabilization control problem with the actuator dynamics. A finite-time control law is derived using the basic double FTSM manifold. Then, an adaptive finite-time control law is proposed to address the issue without prior knowledge of the time matrix. Furthermore, the Lyapunov-based theory is used to prove the finite-time convergence of the closed-loop system. Finally, the simulations are presented to demonstrate the effectiveness of the proposed control law. The results show the high-precision performance of the attitude control and the bounded estimation of the time matrix of the actuator by the proposed scheme.

## Appendix A

**Proof** **of** **Theorem 1.** The Lyapunov stabilization theory is used in the proof, which has three parts: (1) Convergence of the sliding mode manifold S in finite time Define the following Lyapunov function candidate $V_{1}$.

(A1) $$V_{1}=\frac{1}{2}S^{T}PS$$

Taking the time derivative of $V_{1}$ and combining with Equation (13) yields

(A2) $${\dot{V}}_{1}=S^{T}P\dot{S}=S^{T}\left[v+TJ\left[\begin{array}{l} J^{-1}\left(-{\dot{w}}^{\times }Jw-w^{\times }J\dot{w}\right)+\alpha_{1}{\ddot{q}}_{e}\\ +\alpha_{2}a^{\prime \prime }(q_{e})+\beta_{1}\dot{\sigma }+\beta_{2}b^{\prime }(\sigma )+J^{-1}\dot{d} \end{array}\right]-u\right]$$

Using the control law (14) and Assumption 2–3, we have

(A3) $$\begin{array}{ll} {\dot{V}}_{1} & =-S^{T}\left[K_{1}S+K_{2}\frac{S}{\left\|S\right\|}+T\dot{d}\right]\\ & \leq -K_{1}S^{T}S-S^{T}K_{2}\frac{S}{\left\|S\right\|}+\lambda_{\max}\left(T\right)\left\|S\right\|{\dot{d}}_{\max} \end{array}$$

With the choices of $K_{1}$, $K_{2}$ in Equation (15), the following inequalities can be obtained

(A4) $${\dot{V}}_{1}\leq -K_{1}{\left\|S\right\|}^{2}-\varsigma \left\|S\right\|$$

that is

(A5) $${\dot{V}}_{1}+\lambda_{1}V_{1}+\lambda_{2}V_{1}^{\frac{1}{2}}\leq 0$$

where $\lambda_{1}=2K_{1}$, $\lambda_{2}=\sqrt{2}\varsigma$. According to Lemma 1, because $\lambda_{1}$, $\lambda_{2}$ are a positive scalars, the sliding manifold S can converge to origin in finite time, and the convergence time can be given as

(A6) $$t_{1}\leq \frac{2}{\lambda_{1}}\ln\frac{\lambda_{1}V_{2}^{\frac{1}{2}}(0)+\lambda_{2}}{\lambda_{2}}$$

(2) Convergence of sliding manifold mode σ in finite time To prove that σ can converge to σ=0 in finite time after the sliding mode manifold S=0 is achieved, a Lyapunov function candidate is constructed as

(A7) $$V_{2}=\frac{1}{2}\sigma^{T}\sigma$$

Taking the time derivative of $V_{2}$, and using Lemma 2 yield

(A8) $${\dot{V}}_{2}=\sigma^{T}\dot{\sigma }=-\sigma^{T}\left[\beta_{1}\sigma +\beta_{2}\sigma^{\frac{b_{1}}{b_{2}}}\right]\leq -\beta_{1}\sigma^{T}\sigma -\beta_{2}{\left(\sum_{i=1}^{3}{\left|\sigma_{i}\right|}^{2}\right)}^{\frac{\frac{b_{1}}{b_{2}}+1}{2}}$$

that is

(A9) $${\dot{V}}_{1}+\lambda_{3}V_{2}+\lambda_{4}V_{2}^{\kappa }\leq 0$$

where $\lambda_{3}=2\beta_{1}$, $\lambda_{4}=\beta_{2}2^{\frac{\frac{b_{1}}{b_{2}}+1}{2}}$, $\kappa =\frac{\frac{b_{1}}{b_{2}}+1}{2}$. Using Lemma 1, the sliding manifold σ converges to origin in finite time, and the convergence time satisfies

(A10) $$t_{2}\leq \frac{1}{\lambda_{3}(1-\kappa )}\ln\frac{\lambda_{3}V_{2}^{1-\kappa }(0)+\lambda_{4}}{\lambda_{4}}$$

(3) Convergence of attitude quaternion $q_{e}$ and angular velocity w in finite time To prove the convergence of $q_{e}$, a Lyapunov function candidate is defined as

(A11) $$V_{3}=\frac{1}{2}q_{e}^{T}q_{e}$$

Taking the derivative and using the sliding manifold σ=0, we have

(A12) $${\dot{V}}_{3}=q_{e}^{T}{\dot{q}}_{e}=-q_{e}^{T}\frac{1}{2}\left(q_{e0}I_{3}+q_{e}^{\times }\right)\left(\alpha_{1}q_{e}+\alpha_{2}q_{e}^{\frac{a_{1}}{a_{2}}}\right)=-\frac{1}{2}q_{e0}\left(\alpha_{1}q_{e}^{T}q_{e}+\alpha_{2}q_{e}^{T}q_{e}^{\frac{a_{1}}{a_{2}}}\right)$$

According to Lemma 2, the following inequality can be obtained

(A13) $${\dot{V}}_{3}\leq -\frac{1}{2}q_{e0}\left(\alpha_{1}q_{e}^{T}q_{e}+\alpha_{2}{\left(\sum_{i=1}^{3}{\left|q_{ei}\right|}^{2}\right)}^{\frac{\frac{a_{1}}{a_{2}}+1}{2}}\right)$$

Thus, using Lemma 1 and Assumption 1, we can conclude that the $q_{e}$ converges to orgin in finite time, and the convergence time satisfies

(A14) $$t_{3}\leq \frac{1}{\lambda_{5}(1-\tau )}\ln\frac{\lambda_{5}V_{3}^{1-\tau }(0)+\lambda_{6}}{\lambda_{6}}$$

where $\lambda_{5}=\alpha_{1}q_{e0}$, $\lambda_{6}=\alpha_{2}q_{e0}2^{\frac{a_{1}}{2a_{2}}}$, $\tau =\frac{\frac{a_{1}}{a_{2}}+1}{2}$. Because the sliding manifold σ=0 is guaranteed, the angular velocity w also converges to origin along the sliding manifold σ in finite time. Synthesizing the above analysis, the total stabilization time can be given as

(A15) $$T\leq t_{1}+t_{2}+t_{3}$$

The proof is completed. □

## Appendix B

**Proof** **of** **Theorem 2.** The proof is similar to that of Theorem 1, and has three parts as well: (1) Convergence of sliding mode manifold S Construct a Lyapunov function candidate as

(A16) $${\dot{V}}_{1}=\frac{1}{2}S^{T}PS+\frac{1}{2}V{\left(\tilde{T}\right)}^{T}MV(\tilde{T})$$

Differentiating it and combining $\dot{\tilde{T}}=-\dot{\hat{T}}$, the control law (16), and the adaptive law (18), we have

(A17) $$\begin{array}{ll} {\dot{V}}_{1} & =S^{T}P\dot{S}+V(\tilde{T}{)}^{T}MV(\dot{\tilde{T}})\\ & =S^{T}\left\{\tilde{T}\left[\begin{array}{l} \left(-{\dot{w}}^{\times }Jw-w^{\times }J\dot{w}\right)+\\ J\left(\alpha_{1}{\ddot{q}}_{e}+\alpha_{2}a^{\prime \prime }(q_{e})+\beta_{1}\dot{\sigma }\;+\beta_{2}b^{\prime }(\sigma )\right) \end{array}\right]-K_{1}S-K_{2}\frac{S}{\left\|S\right\|}+T\dot{d}\right\}-V(\tilde{T}{)}^{T}MV(\dot{\hat{T}})\\ & =-S^{T}\left[K_{1}S+K_{2}\frac{S}{\left\|S\right\|}-T\dot{d}\right]+V(\tilde{T}{)}^{T}V(\hat{T})\\ & =-S^{T}\left[K_{1}S+K_{2}\frac{S}{\left\|S\right\|}-T\dot{d}\right]-V(\tilde{T}{)}^{T}V(\tilde{T})+V(\tilde{T}{)}^{T}V(T) \end{array}$$

Using Young’s inequality, the following inequality can be obtained

(A18) $$V\left(\tilde{T}{)}^{T}V(T)\leq \frac{1}{2}\eta V{\left(\tilde{T}\right)}^{T}V(\tilde{T})+\frac{1}{2\eta }V(T)V(T\right)$$

with 0<η<1. Substituting Equation (A18) and choosing $K_{1}$, $K_{2}$ satisfying Equation (17), ${\dot{V}}_{1}$ is bounded as

(A19) $$\begin{array}{ll} {\dot{V}}_{1} & \leq -K_{1}{\left\|S\right\|}^{2}-V(\tilde{T}{)}^{T}V(\tilde{T})+\frac{1}{2}\eta V(\tilde{T}{)}^{T}V(\tilde{T})+\frac{1}{2\eta }V{\left(T\right)}^{T}V(T)\\ & =-K_{1}{\left\|S\right\|}^{2}-\left(1-\frac{1}{2}\eta \right)V{\left(\tilde{T}\right)}^{T}V(\tilde{T})+\frac{1}{2\eta }V{\left(T\right)}^{T}V(T) \end{array}$$

According to Assumption 3, $\frac{1}{2\eta }V{\left(T\right)}^{T}V(T)>0$ holds. Thus, we have

(A20) $${\dot{V}}_{1}\leq -2\xi V_{1}+\varphi$$

where $\xi =\min\left\{K_{1},\left(1-\frac{1}{2}\eta \right)\right\}$, $\varphi =\frac{1}{2\eta }V{\left(T\right)}^{T}V(T)$ It can be concluded that $V_{1}$ is uniformly ultimately bounded with ***S*** and $V(\tilde{T})$. More precisely, ***S*** and $V(\tilde{T})$ converge to the region [20].

(A21) $$\left\|S\right\|\leq \varepsilon ,\;\left\|V(\tilde{T})\right\|\leq \varepsilon$$

in finite time, where $\varepsilon >\sqrt{\frac{\varphi }{\xi }}=\sqrt{\frac{1}{2\eta \xi }V{\left(T\right)}^{T}V(T)}$. (2) Convergence of sliding manifold σ For any sliding mode variable $S_{j}\left(j=1,2,3\right)$ in the region ε, we have $\left|S_{j}\right|\leq \varepsilon$. Then the sliding mode manifold S defined in (10) can be written as

(A22) $$\begin{array}{cc} {\dot{\sigma }}_{j}+\beta_{1}\sigma_{j}+\beta_{2}\sigma_{j}^{\frac{b_{1}}{b_{2}}}=\chi_{j} & \left|\chi_{j}\right|\leq \varepsilon_{1} \end{array}$$

Then Equation (A22) can be rewritten in the following two forms

(A23) $${\dot{\sigma }}_{j}+\left(\beta_{1}-\frac{\chi_{j}}{\sigma_{j}}\right)\sigma_{j}+\beta_{2}\sigma_{j}^{\frac{b_{1}}{b_{2}}}=0$$

(A24) $${\dot{\sigma }}_{j}+\beta_{1}\sigma_{j}+\left(\beta_{2}-\frac{\chi_{j}}{\sigma_{j}^{\frac{b_{1}}{b_{2}}}}\right)\sigma_{j}^{\frac{b_{1}}{b_{2}}}=0$$

From Equation (A23), if $\beta_{1}-\frac{\chi_{j}}{\sigma_{j}}>0$, a similar structure to the proposed sliding mode manifold S is kept, and a similar stabilization proof to the second part of Theorem 1 can be given, which is omitted here. Furthermore, the $\sigma_{j}$ converges to the region

$$\left|\sigma_{j}\right|\leq \frac{\chi_{j}}{\beta_{1}}\leq \frac{\varepsilon }{\beta_{1}}$$

in finite time. By similar analysis for Equation (A24), the $\sigma_{j}$ also converges to the region

$$\left|\sigma_{j}\right|\leq {\left(\frac{\chi_{j}}{\beta_{2}}\right)}^{\frac{b_{2}}{b_{1}}}\leq {\left(\frac{\varepsilon }{\beta_{2}}\right)}^{\frac{b_{2}}{b_{1}}}$$

in finite time. Finally, the $\sigma_{j}$ converges to the region

(A25) $$\left|\sigma_{j}\right|\leq \delta$$

in finite time, where $\delta =\min\left\{\frac{\varepsilon }{\beta_{1}},{\left(\frac{\varepsilon }{\beta_{2}}\right)}^{\frac{b_{2}}{b_{1}}}\right\}$. (3) Convergence of attitude quaternion $q_{e}$ and angle velocity w For any sliding mode variable $\sigma_{j}\left(j=1,2,3\right)$ in the region δ, we have $\left|\sigma_{j}\right|\leq \delta$. Then the sliding mode manifold σ defined in (6) can be written as

(A26) $$\begin{array}{cc} w_{j}+\alpha_{1}q_{ej}+\alpha_{2}q_{ej}^{\frac{a_{1}}{a_{2}}}=\nu_{j} & \left|\nu_{j}\right|\leq \delta \end{array}$$

Similar to the second part of Theorem 2, Equation (A26) can be rewritten in the following two forms

(A27) $$w_{j}+\left(\alpha_{1}-\frac{\nu_{j}}{q_{ej}}\right)q_{ej}+\alpha_{2}q_{ej}^{\frac{a_{1}}{a_{2}}}=0$$

(A28) $$w_{j}+\alpha_{1}q_{ej}+\left(\alpha_{2}-\frac{\nu_{j}}{q_{ej}^{\frac{a_{1}}{a_{2}}}}\right)q_{ej}^{\frac{a_{1}}{a_{2}}}=0$$

From Equation (A27), if $\alpha_{1}-\frac{\nu_{j}}{q_{ej}}>0$, a similar stabilization proof to the third part of Theorem 1 can be given, which is omitted here. Furthermore, the $q_{ej}$ converges to the region

$$\left|q_{ej}\right|\leq \frac{\nu_{j}}{\alpha_{1}}\leq \frac{\delta }{\alpha_{1}}$$

in finite time. By similar analysis for Equation (A28), the $q_{ej}$ also converges to the region

$$\left|q_{ej}\right|\leq {\left(\frac{\nu_{j}}{\alpha_{2}}\right)}^{\frac{a_{2}}{a_{1}}}\leq {\left(\frac{\delta }{\alpha_{2}}\right)}^{\frac{a_{2}}{a_{1}}}$$

in finite time. Finally, the $q_{ej}$ converges to the region

(A29) $$\left|q_{ej}\right|\leq \Delta$$

in finite time, where $\Delta =\min\left\{\frac{\delta }{\alpha_{1}},{\left(\frac{\delta }{\alpha_{2}}\right)}^{\frac{a_{2}}{a_{1}}}\right\}$. Moreover, from Equation (A26), w converges to the region in finite time.

(A30) $$\left|w_{j}\right|\leq \alpha_{1}\left|q_{ej}\right|+\alpha_{2}q_{ej}^{\frac{a_{1}}{a_{2}}}+\delta \leq 3\delta$$

The proof is completed. □
